# Interaction of single and multi wall carbon nanotubes with the biological systems: tau protein and PC12 cells as targets

**DOI:** 10.1038/srep26508

**Published:** 2016-05-24

**Authors:** Hojjat Alizadeh Zeinabad, Alireza Zarrabian, Ali Akbar Saboury, Ali Mohammad Alizadeh, Mojtaba Falahati

**Affiliations:** 1 Department of nanotechnology, Faculty of Advance Science and Technology, Pharmaceutical Sciences Branch, Islamic Azad University (IAUPS), Tehran, Iran; 2 Institute of Biochemistry and Biophysics, University of Tehran, Tehran, Iran; 3 Cancer Research Center, Tehran University of Medical Sciences, Tehran, Iran

## Abstract

Subtle changes in the structure of nanoparticles influence their surface tension and corresponding interaction with cells and proteins. Here, the interaction of the single wall carbon nanotube (SWCNT) and multiwall carbon nanotube (MWCNT) with different surface tension with tau protein was evaluated using a variety of techniques including far and near circular dichroism, fluorescence spectroscopy, dynamic light scattering, Zeta potential, and TEM evaluation. Also the cytotoxicity of SWCNT and MWCNT on the PC12 cell line as a model of nervous system cell line was investigated by the MTT, LDH, acridine orange/ethidium bromide staining, flow cytometry, caspase 3 activity, cell and membrane potential assays. It was observed that SWCNT induced more structural changes of tau protein relative to MWCNT/tau protein interaction. It was also revealed that SWCNT and MWCNT impaired the viability and complexity of PC12 cells in different modes of cytotoxicity. Analysis of cellular outcomes indicated that MWCNT in comparison with SWCNT resulted in induction of necrotic modes of cell death, whereas apoptotic modes of cell death were activated in SWCNT-incubated cells. Together these findings suggest that surface tension may be used to determine how nanoparticle structure affects neurotoxicity and protein conformational changes.

Carbon nanotubes (CNTs) present several unique chemical, thermal, optical, mechanical, electrical and structural properties that make them ideal candidate in biomedical application for the treatment of wide range of disorders^1^. They also served as the noninvasive method for monitoring of chemical properties of the human body^2^. CNTs are great candidate in medical research and are being highly used in the fields of targeted drug delivery and, several disease treatments and monitoring of cells^3^,^4^. One of the main disadvantages of CNTs is the lack of solubility and the low biocompatibility in the physiological media. CNTs have been functionalized with the different hydrophilic moieties to overcome these problems^5^. Application of CNTs in biomedical area is hampered by their biodistribution and kinetics of CNTs. These parameters are affected by nanoparticle characteristics such as shape, size and surface functionalization. High surface area of CNTs can also lead to their intrinsic toxicity, and may be the main challenging reason for their harmful effects in the biomedical applications^6^,^7^,^8^,^9^,^10^,^11^. The toxicity of CNTs can also be affected by the diameter of nanotubes based on single or multi wall structures. Unique properties of single wall carbon nanotube (SWCNT) and multi wall carbon nanotube (MWCNT) result in the different toxicity to the cells and the structural changes of proteins^12^,^13^,^14^,^15^,^16^,^17^,^18^. However, compared to other studies dealing with synthesis, characterization, and applications of SWCNT and MWCNT, to date, only a few reports have been investigated the different CNTs effects (single and/or multi wall) on the protein structure and the cell morphology, and these reports seem to be conflicting. For instance, it has been demonstrated that CNTs may result in reduction of keratinocyte cell viability due to oxidative stress, SWCNT is more toxic than MWCNT in macrophages, and inhaled MWCNTs can switch the several toxicological pathways in respiratory epithelium^19^,^20^,^21^. In contrast, Huczko *et al*., Kam *et al*., Fiorito *et al*., Nagai *et al*. and Toyokuni *et al*. reported CNT stimulated negligible risk of cytotoxicity^22^,^23^,^24^,^25^,^26^. It has been reported that interaction of SWCNT with horseradish peroxidase and chicken egg white lysozyme doesn’t change on the protein structure^27^. The interaction of MWCNT with catalase revealed that the activity of protein was inhibited, and secondary and tertiary structures of protein were changed^28^. Also interaction of CNT with different proteins such as hydrolase and albumin had been investigated and relation of nanoparticle surface curvature and protein conformational changes had been studied^29^,^30^,^31^.

The solution surface tension is a descent criterion which correlates with stability and function of proteins and nanoparticles. Nanoparticles with different geometry alter the surface tension of solutions at the air/water interface. Thus, surface tension can be applied to deduce the nanoparticle-induced changes of protein structure and specific destructive action on the cell.

Therefore, this study was carried out to determine the effects of SWCNT and MWCNT on tau protein and PC12 cells as targets to investigate the effect of different CNTs on nervous system *in vitro*. Tau protein is a member of protein family known as Microtubule-Associated Proteins (MAPs). This protein possesses a very low content of the secondary structure and the hydrophobic patches, and considered to be a “natively unfolded” protein^32^. In this study we have investigated the interaction of SWCNT and MWCNT with tau protein as a model of nervous system protein by far and near UV-circular dichroism (CD) and fluorescence spectroscopy. PC12 cells exhibited key characteristics of dopaminergic neuronal cells, and have been widely used as a model for neuronal cells studies. We used this model to investigate the correlation between surface tension of SWCNT and MWCNT and cytotoxicity on PC12 cells as a model of nervous system cell by MTT, LDH, flow cytometry, mitochondrial and cell membrane potential assays. As a result, we demonstrated a strong correlation between the surface tension of CNTs solutions and the ability of these nanoparticles to interact with protein and cell. This research can be used to rationally design a nanocarrier for drug delivery design and therapy.

## Results

### Characterization of SWCNT and MWCNT

SWCNT and MWCNT along with their data sheets were purchased from Neutrino Company which is one of Iran’s main suppliers in nanotechnology field. Briefly morphology, size distribution and properties of CNTs are given in Table 1.

Of the enchanting features of CNTs, the study of optical phenomena is potentially receiving increasing attention. Now it’s known that the absorbance properties of SWCNT and MWCNT, normally carries a number of crucial factors. From long to short wavelength, in UV-vis region, characteristic of individual CNTs of unique diameters, are normally revealed.

**Figure 1:**
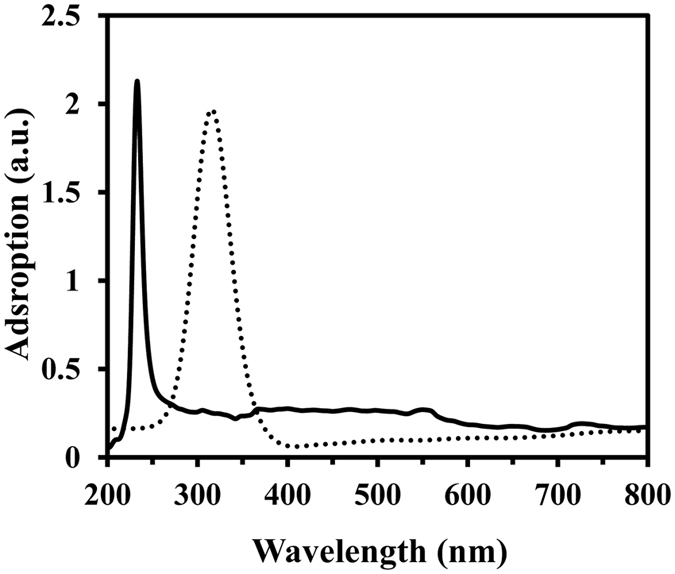
UV–vis absorbance spectra of SWCNT and MWCNT samples. UV–vis absorbance spectra of SWCNT (continues) and MWCNT (dashed) suspensions, with a concentration of 0.4 mg/mL in ethanol.

At remarkably higher energy, in the UV region, two unique bands are seen for SWCNT and MWCNT (Fig. 1). This is due to different plasmon resonances of free electron of the nanotubes π electrons, at around 233 and 312 nm for SWCNT and MWCNT, respectively (Fig. 1). With an increase in nanotube diameter resulting in a corresponding red-shift to higher wavelength a clear relationship between the absorption energy of the π electrons and mean CNT diameter is observed which verifies the previous report^33^. It can also be assumed that the sharpness of the peaks is due to a relatively narrow size distribution. This could be due to dispersion, approximately identical tube lengths, and diameter.

**Figure 2:**
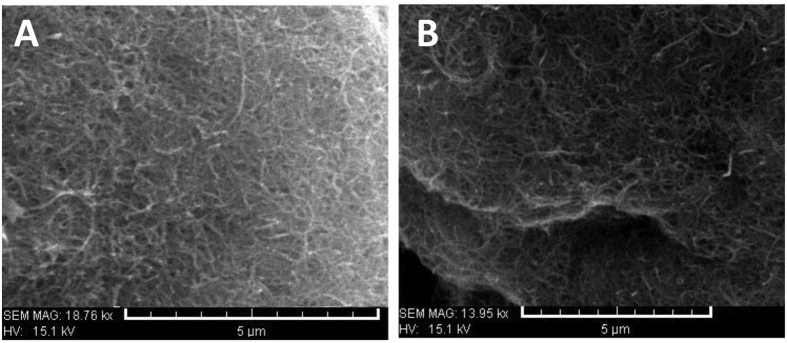
SEM images of CNTs. SEM images of SWCNT (**A**) and MWCNT (**B**).

Observing Fig. 2(A,B) demonstrates typical SEM images of CNTs with high purity. The SEM images show that SWCNT (Fig. 2A) and MWCNT (Fig. 2B) provide microns long with uniform diameters and also, the SEM observations indicate that the samples are clean.

**Figure 3:**
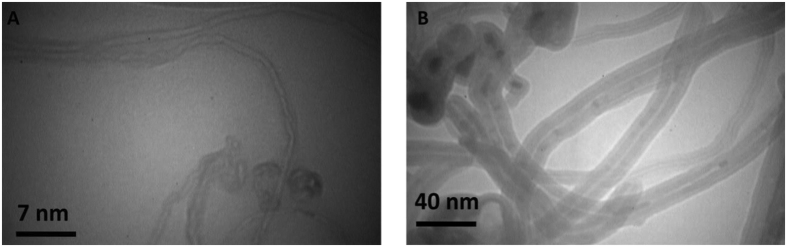
TEM images of CNTs. TEM images of SWCNT (**A**) and MWCNT (**B**).

Figure 3 shows TEM images of SWCNT and MWCNT dispersed in ethanol. It is evident, from the images that all the CNTs provide hollow and tubular shapes. TEM images specify that the CNTs contain high purity, uniform size distribution, and no deformity in the structure. The dispersion conditions of SWCNT and MWCNT were also visualized using TEM analysis. Based on the TEM images of Fig. 3(A,B), it can be concluded that the SWCNT and MWCNT dispersed in ethanol present diameter of about 1–2 nm and 10–20 nm, respectively.

### Dynamic light scattering studies

Nanoparticles dispersion and their agglomeration can induce a great effect in nanomaterial toxicity. Therefore it is crucial to determine physical and chemical properties of CNT in physiological solution. Dynamic light scattering (DLS) is one sensitive method for providing information of size and charge distribution for sphere shaped nanomaterials and nanotubes^34^,^35^. Therefore the DLS data could be utilized to approximately evaluate variation of size distribution for CNTs. The measurement of DLS and Zeta potential was conducted with the two kinds of SWCNT and MWCNT dispersing in PBS buffer or cell culture medium. The size distribution and the properties of CNTs are given in Table 1. Apparent CNTs size increased in both PBS and cell culture medium. It was observed a size distribution of SWCNT with ~40 and ~61 nm, and MWCNT with ~160 and ~171 nm at 20 μg/mL in PBS and cell culture medium, respectively (Table 2).

Zeta potential measurement showed that SWCNT and MWCNT had identical Zeta potential values around −4 mV when dispersing in PBS buffer solution, and around −18 mV when dispersing in cell culture medium (Table 3). It is indicative that the diameter enhancement of CNTs did not affect their charge distribution and the dispersion stability in PBS and cell culture medium. When CNTs are dispersing in the cell culture medium, Zeta potentials of the two kinds of CNTs were approximately *−*18 mV (Table 3), which is almost identical to that of cell culture medium (*−*17 mV). The data revealed that serum protein molecules of cell culture medium were absorbed onto two types of CNTs surfaces, and when the diameter increased; Zeta potential value exhibited a similar tendency which implied that CNTs with unfunctionalized surface degree had identical surface charges. Moreover, the average hydrodynamic size for SWCNT and MWCNT dispersing in the cell culture medium was larger than dispersing in PBS, which supports adsorption of serum protein onto the CNTs surface.

Taken in all, the average charge distribution of the SWCNT and MWCNT remained almost identical when dispersing in PBS. Dispersed SWCNT and MWCNT also exhibited average charge distribution in a closed level in the cell culture medium.

### Surface tension of the CNTs solution

The hydrophobic effect can be easily monitored through changes in the surface tension for an air/water interface. In the aqueous solution containing nanoparticles and proteins, the changes in their shape, size, and geometry, alters the surface tension of solutions at the air/water interface. In other words, evaluating changes in the surface tension of the air-water interface enable to detect the efficiency of the assembly and disassembly of materials such as proteins and nanoparticles into the surface. The alteration of surface tension of solutions has also different impact on conformational changes of materials. Being driven by the thermodynamic potential for decreasing surface tension, the hydrophobic nanoparticles and hydrophobic patches of proteins may move down from the hydrophilic phase of the system and may associate with each other by hydrophobic-hydrophobic interactions and lead to material aggregation. Hydrophilic surfaces bind to surface of proteins and hydrophilic nanoparticles more firmly than hydrophobic surfaces. The main reason for this is the increase in interaction between hydrophilic surfaces and hydrophilic moiety of proteins and nanoparticle that are exposed during interaction. Interfacial water is then strongly bound to the contacting hydrophilic surfaces compared to same process on hydrophobic surfaces with weaker water binding^36^,^37^.

**Figure 4:**
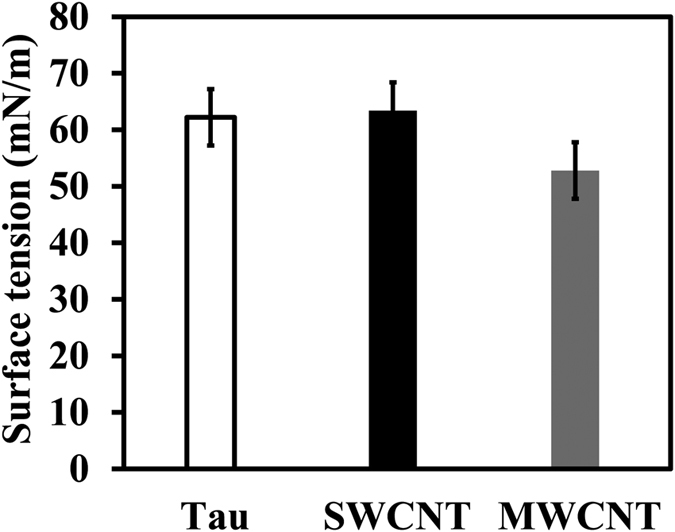
Surface tension measurements of tau protein, SWCNT and MWCNT. Surface tension changes of solutions containing tau protein, SWCNT and MWCNT at the air-water interface.

Figure 4 shows that the surface tensions of tau protein, SWCNT and MWCNT solutions were approximately 62.2, 63.4 and 52.8 mN/m, respectively. The amount of surface tension of SWCNT and MWCNT in PBS relative to cell culture medium was almost identical (data not shown). Such a significant decrease in surface tension of MWCNT was accompanied by its unique geometry and increase in hydrophobic content of the structure. Furthermore, significant differences in the surface tension of CNTs molecules are reflected in the geometry of SWCNT and MWCNT and corresponding surface polarity. For tau protein, the surface tension was almost similar to SWCNT, reflecting more polar-polar interactions relative to MWCNT/tau protein. On the other hand, the surface tension of MWCNT was lower than both tau protein and SWCNT, indicating that the MWCNT is more hydrophobic than SWCNT and tau protein. Surface tension data variation is probably due to the different radius of CNTs and distribution number of carbon atoms.

### Circular dichroism studies of SWCNT/tau and MWCNT/tau protein

**Figure 5:**
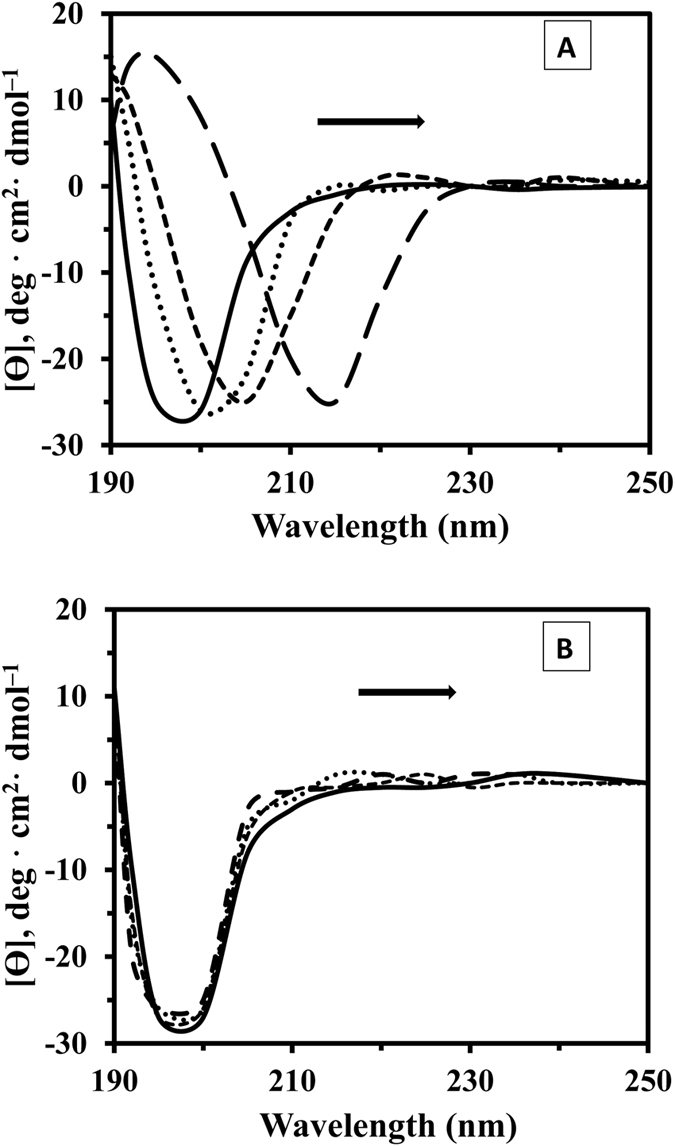
Far UV-CD spectra of tau protein during incubation with SWCNT and MWCNT. Far-UV CD spectra, recorded for SWCNT-tau protein (**A**) and MWCNT-tau protein (**B**) at the tau protein concentration of 200 μg/mL (phosphate buffer 20 mM, pH 7.8) and CNTs concentrations of 0, 10, 50, and 100 μg/mL at 25 °C. At the onset of tau protein one minimum at 198 nm indicates random coil structure. After SWCNT incubation, appearance of a new minimum at approximately 218 nm indicates cross β–sheet structure while in the case of MWCNT/tau protein secondary structural changes was not happen.

Far UV-CD (190–250 nm) and the near UV-CD (240–320 nm) spectra can sensitively monitor secondary and tertiary conformation changes of proteins upon interaction with the nanoparticles, respectively^37^. The far UV-CD of tau exhibits a minimum close to 195 nm corresponding to a mostly random coil structure of tau soluble protein^38^. The spectrum of the tau sample after raising concentration of SWCNT exhibits a significant shift of the minimum to higher wavelengths. Such a broad minimum at ∼218 nm is a typical characteristic of beta sheet structure of tau protein (Fig. 5A). The increase of β-sheet content indicates that the binding of tau and SWCNT induces the protein folding and more compact structure.

As shown in Fig. 5B, the band intensity and position of protein had not changed, suggesting that binding of MWCNT has not altered the secondary structure of tau protein. This study shows that SWCNT exhibited greater interactions with tau protein structure, resulting in more pronounced conformational changes and corresponding denaturation.

**Figure 6:**
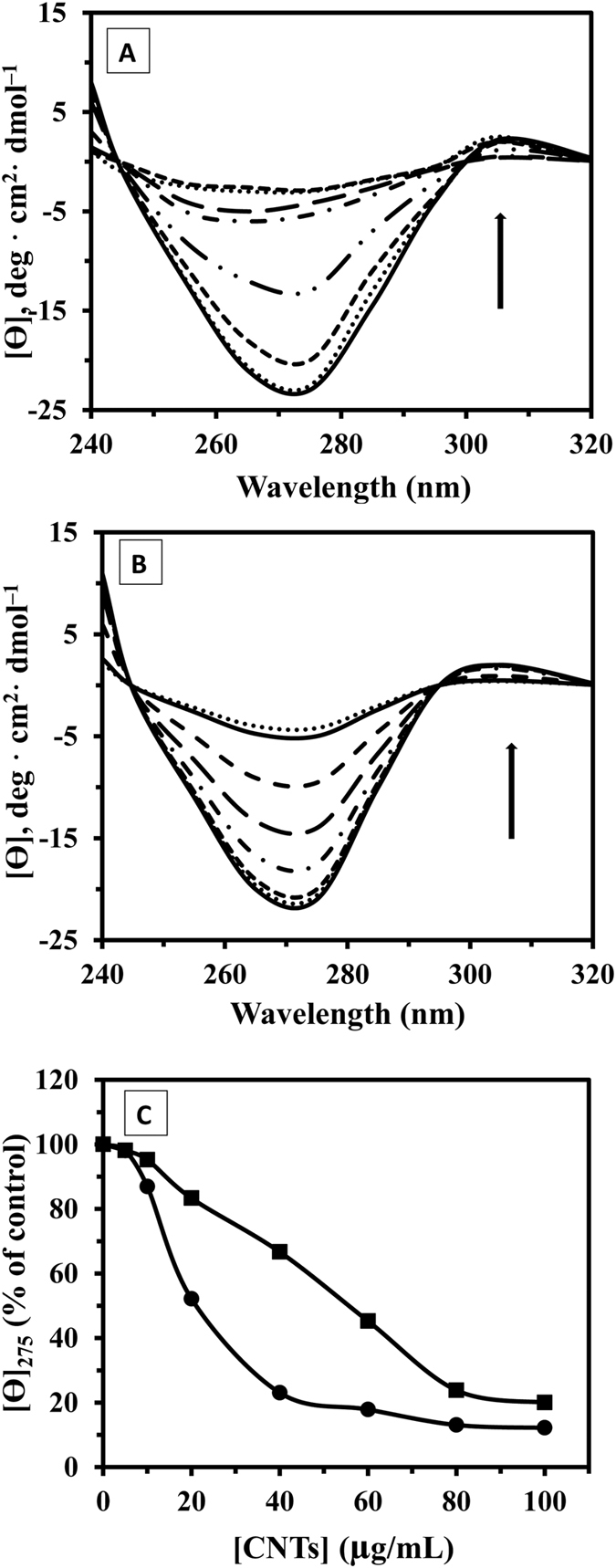
Near UV-CD spectra of tau protein during incubation with SWCNT and MWCNT. Near UV-CD spectra, recorded for SWCNT-tau protein (**A**) and MWCNT-tau protein (**B**) at the tau protein concentration of 500 μg/mL (phosphate buffer 20 mM, pH 7.8) and CNTs concentrations of 0, 5 10, 20, 40, 60, 80, and 100 μg/mL at 25 °C. The DC_50_ for SWCNT/tau and MWCNT/tau interactions was 21.7 and 53.2 μg/mL, respectively (**C**).

The stability of the tertiary structure of tau protein upon interaction with CNTs was also monitored by near UV-CD to obtain complementary information about the structural changes mechanism of proteins. Figure 6A–C demonstrates tertiary structural changes of tau protein after titration of raising concentration of CNTs. The results revealed that upon raising the CNTs concentration, the magnitude of the near-UV spectrum of tau protein was reduced. This reduction was more pronounced for SWCNT/tau interaction compared to MWCNT/tau, indicating a more significant disruption of the tertiary structure of tau protein in the presence of SWCNT relative to MWCNT (Fig. 6A,B).

Half-denaturation concentration (DC_50_) of CNTs is a useful indicator of the ligand-induced conformational changes and generally stability of protein. The DC_50_ for SWCNT/tau and MWCNT/tau interactions was 21.7 and 53.2 μg/ml, respectively (Fig. 6C). It shows the stability of tau protein was dramatically reduced with binding to SWCNT. SWCNTs were capable of inducing greater loss of structure as compared to MWCNTs.

It is hypothesized that the relatively larger surface tension afforded by the smaller CNTs (SWCNT) allows more interaction between the nanoparticle surface and protein structure, while a lager CNTs (MWCNT) has a smaller surface tension, resulting interactions and conformational changes. Therefore, as a result of tau protein conformational changes, it may be concluded that SWCNTs are usually more toxic than MWCNTs. This suggests that conformational changes of tau protein may be retarded simply by using larger nanotubes as drug carrier material.

Fluorescence study was also conducted to explore the exact mechanism of interaction between CNTs and tau proteins.

### Fluorescence Quenching of tau protein by CNTs

**Figure 7:**
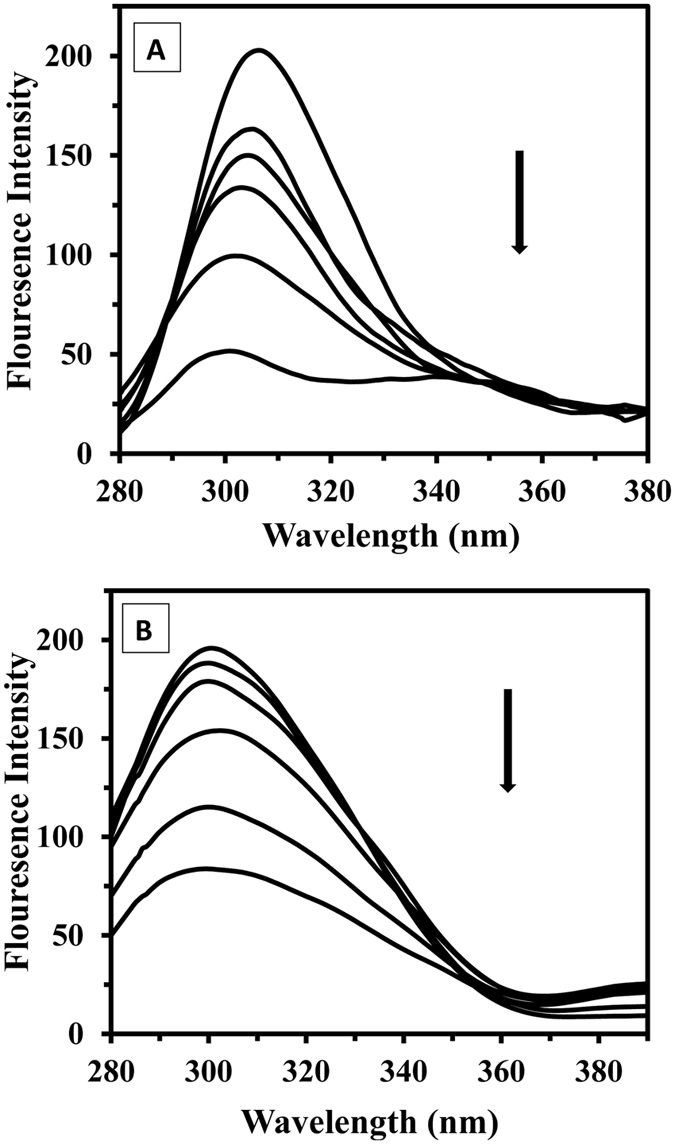
Fluorescence studies of tau protein upon incubation with SWCNT and MWCNT. Effects of SWCNT on the fluorescence spectrum of tau protein (**A**) and MWCNT on the fluorescence spectrum of tau protein (**B**) at the tau protein concentration of 50 μg/mL (phosphate buffer 20 mM, pH 7.8) and CNTs concentrations of 0, 5, 10, 20, 50, and 100 μg/mL at 25 °C.

Fluorescence spectroscopy is a very powerful method for studying ligand-protein interactions^39^. This technique provides rapid and sensitive analysis for determining the binding of different chemical compounds to proteins. Generally, the intrinsic fluorescence of a protein is contributed to by three fluorophores present in the protein structure, i.e., the tryptophan (Trp), tyrosine (Tyr) and phenylalanine (Phe) residues^39^. Tau protein emits the strong fluorescence peaks of 307 nm contributed to presence of Tyr residues (excitation wavelengths of 274 nm). When the tau protein solution was titrated with increasing amounts of SWCNT and MWCNT, its fluorescence intensities at 307 nm were significantly quenched (Fig. 7A,B). The quenching of the tau protein fluorescence upon interaction with CNTs suggests that the fluorophore of tau protein was positioned in a hydrophobic environment.

**Figure 8:**
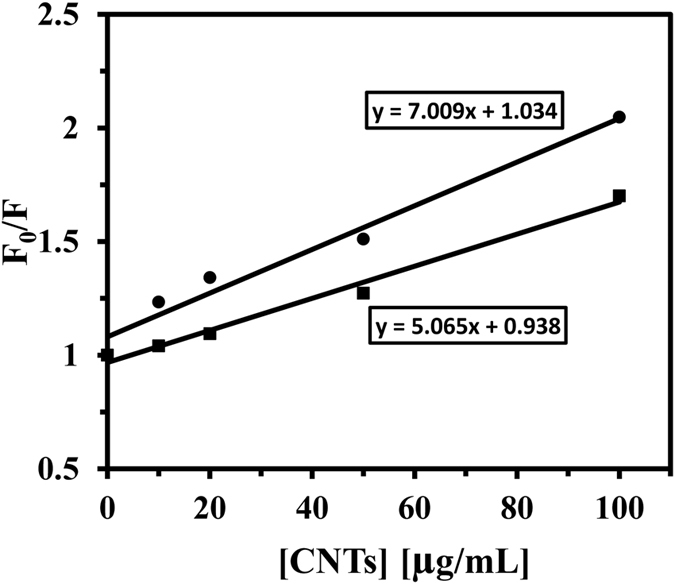
Stern–Volmer plot. Stern–Volmer plot for the binding of SWCNT with tau protein (⚫) and MWCNT with tau protein (◾) at 25 °C.

When the concentration of SWCNT increased to 100 μg/mL, the maximum emission wavelength of the tau protein showed a blue-shift of about 6 nm, which indicates a reduction of the microenvironment polarity has occurred, and also hydrophobic part of protein such as tyrosine residue was buried (Fig. 7A). No obvious shift of the maximum emission wavelength of the tau protein with interaction of MWCNT was observed, indicating MWCNT could not cause the change of tau protein conformation (Fig. 7B). In order to understand the difference between the interaction of SWCNT and MWCNT with proteins, the Stern-Volmer curves of the tau protein were plotted and the results are shown in Fig. 8. The Stern-Volmer plots of tau protein with SWCNT and MWCNT were different, indicating different CNTs might have non-identical interaction mode with tau protein, which is accompanied by non-specific interaction of protein and CNTs.

The fluorescence quenching data analyzed by the Stern–Volmer Eq. 1^39^:


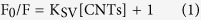


where, F_0_ and F are the steady-state fluorescence intensities in the absence and presence of CNTs, respectively. K_SV_ is the Stern–Volmer quenching constant, and [CNTs] is the concentration of SWCNT and MWCNT. The value of K_SV_ was obtained from the slope of the Stern-Volmer graph and found to be 7.00 and 5.06 mL/μg for SWCNT and MWCNT, respectively (Fig. 8).

We noted that SWCNT displays value of K_SV_ higher than MWCNT assigning further evidence for static quenching (protein-CNT complex formation) of SWCNT relative to MWCNT^37^. The obtained data strongly indicates that SWCNT bind to tau protein stronger than MWCNT to form protein-CNT system^40^. Application of this information to extrapolate effects of such nanotubes in cellular studies however needs further experiments

### SWCNT and MWCNT cytotoxicity on PC12 cells

**Figure 9:**
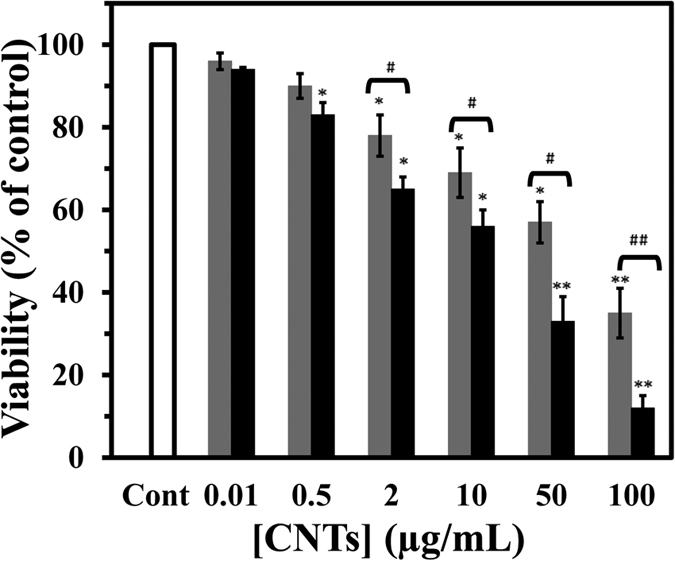
Effect of SWCNT and MWCNT on cell viability of PC12 cell. Cells were incubated with raising concentration of SWCNT (black) or MWCNT (gray) for 48 h at 37 °C. The percentage of cell viability was achieved by the MTT assay as explained in materials and methods. Data are shown as average of three separate experiments and error bars represent standard deviation (SD). *P < 0.05 and **P < 0.01 represents the significant differences between CNTs –treated groups and control. ^#^P < 0.05 and ^##^P < 0.01 represents the significant differences between SWCNT –treated groups and MWCNT –treated groups.

The cytotoxicity of SWCNT and MWCNT on PC12 cells line (rat adrenal pheochromocytoma cells) was determined by the MTT assay for monitoring cell viability. The cells were incubated with various concentrations of SWCNT and MWCNT at 37 °C for 48 h. All measurements were normalized to the untreated cells as control. SWCNT were observed to be more toxic to PC12 cells than MWCNT: extrapolation from the dose-response curve shows that a dose of 22.7 μg/mL of SWCNT inhibited 50% of the PC12 cell population after 48 h, whereas a dose of 65.5 μg/mL MWCNT inhibited 50% of the cell population (Fig. 9). Figure 9 reveals that the SWCNT was more toxic than the MWCNT for the same concentration.

### SWCNT and MWCNT on LDH release *in vitro*

**Figure 10:**
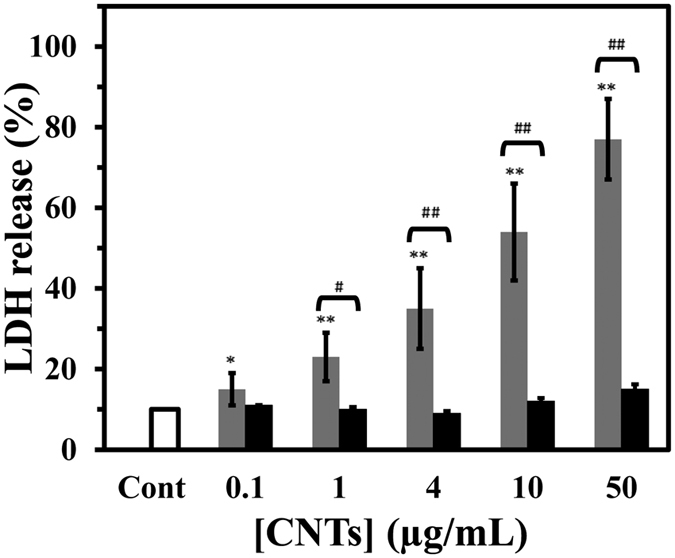
Effect of raising concentration of SWCNT and MWCNT on LDH release of PC12 cell. Cells were treated with raising concentration of SWCNT (black) or MWCNT (gray) for 48 h at 37 °C. The percentage of cell viability was calculated by the LDH assay as explained in materials and methods. Data are shown as average of three separate experiments and error bars represent standard deviation (SD). *P < 0.05 and **P < 0.01 represents the significant differences between CNTs –treated groups and control. ^#^P < 0.05 and ^##^P < 0.01 represents the significant differences between MWCNT –treated groups and SWCNT –treated groups.

CNTs-induced cell membrane leakage was detected by LDH release assay. After 48 h, treatment of PC12 cells with MWCNT resulted in a significant increase in LDH release relative to SWCNT (Fig. 10). After 48 h of treatment with 50 μg/mL MWCNT, LDH release was increased to 7.7 fold relative to control (p < 0.01). Treatment with 4 μg/mL MWCNT increased LDH release to 3.5 fold in comparison with control (p < 0.05), whereas treatment with 1 μg/mL MWCNT was 2.3 fold relative to control. In contrast, LDH determination of cultured media of PC12 cells exposed to various concentrations of SWCNT indicated that no significant difference occurred in relative to control cells.

### Detection of PC12 cell apoptosis or necrosis with SWCNT and MWCNT by fluorescence microscopy

**Figure 11:**
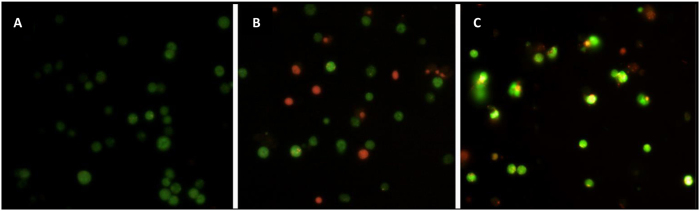
Effect of IC_50_ of SWCNT and MWCNT on PC12 cell apoptosis and necrosis. The mode of cell death [(MWCNT-treated cells (**B**), SWCNT-treated cells (**C**)] (different necrotic or apoptotic cells) was detected by using AO/EB dual staining method in comparison with control cells, which appeared uniformly stained green in color (**A**).

In current experiment the mode of cell death (necrosis versus apoptosis) was detected by using AO/EB dual staining method. In comparison with control cells, which appeared uniformly stained green in color (Fig. 11A–C), apoptotic cells contain bright green and orange condensed bodies in their nuclei representing nuclear DNA fragmentation, and necrotic cells present uniformly orange-stained cell nuclei^41^. Analysis of results obtained in AO/EB dual staining indicated that MWCNT in comparison with SWCNT resulted in activation of necrotic modes of cell death (Fig. 11B), whereas Fig. 11C suggests that apoptotic modes of cell death are induced in SWCNT-exposed PC12 cells.

### Flow cytometric analysis

**Figure 12:**
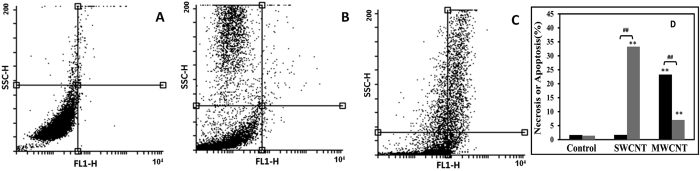
Flow cytometry assay to evaluate cell death. Cells were co-labeled with PI and Annexin V-FITC, and analyzed by flow cytometer. The histograms of untreated control cells (**A**), cells exposed to MWCNT for 48 h (**B**) and cells exposed to SWCNT for 48 h (**C**). The percentage of apoptotic (black) and necrotic (gray) cells after 48 h incubation with SWCNT and MWCNT (**D**). Lower left quadrant shows the control surviving cells. The early apoptosis is presented in the lower right quadrant, and the late apoptosis is presented in the upper right quadrant. The necrotic cells are presented in the upper left quadrant. *P < 0.05 and **P < 0.01 represents the significant differences between CNTs –treated groups and control. ^#^P < 0.05 and ^##^P < 0.01 represents the significant differences between MWCNT –treated groups and SWCNT –treated groups.

To quantitatively obtain insight into different cytotoxicity effect of SWCNT and MWCNT, a display of side light scatter (SSC) versus FL1 was carried out by the flow cytometric analysis (Fig. 12A–C). After incubation of PC12 cells for 48 h, it was found that MWCNT (Fig. 12B) and SWCNT (Fig. 12C) triggered the different extent of cell apoptosis. As anticipated, SWCNT had a substantial increase in apoptotic cells (up to 23.23%), while MWCNT caused a significant increase in necrotic cells (up to 32.38%) (Fig. 12D). In agreement with visual observation, the quantification of apoptosis by CNTs affirmed no significant apoptosis death (1.64%) for MWCNT nanomaterial (Fig. 12D).

### CNTs-induced apoptosis in PC12 cells

**Figure 13:**
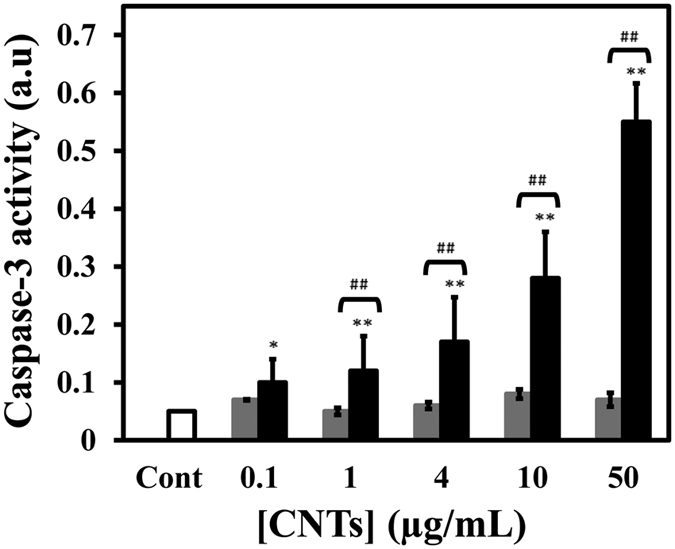
Effect of SWCNT and MWCNT on caspase-3 activity. Caspase-3 was induced following treatment with raising concentration of SWCNT (black) or MWCNT (gray) for 48 h. The activity of caspase-3 was increased in a concentration-dependent manner while this enhancement was almost absent in the case of MWCNT. *P < 0.05 and **P < 0.01 represents the significant differences between CNTs –treated groups and control. ^#^P < 0.05 and ^##^P < 0.01 represents the significant differences between SWCNT –treated groups and MWCNT –treated groups.

Caspase-3 was induced following treatment with raising concentration of SWCNT and MWCNT (Fig. 13). When cells were treated with 0.1, 1, 4, 10 and 50 μg/mL concentrations of SWCNT for 48 h, the activity of caspase-3 was increased in a concentration-dependent manner while this enhancement was almost absent in the case of MWCNT. As shown in Fig. 13, the activation of caspase-3 was significant in SWCNT (50 μg/mL concentration) (p < 0.01). These results suggest that SWCNT-induced apoptosis are associated with the activation of caspase-3, while MWCNT did not activate the apoptosis pathway.

### Effect of SWCNT and MWCNT on cell membrane potential

**Figure 14:**
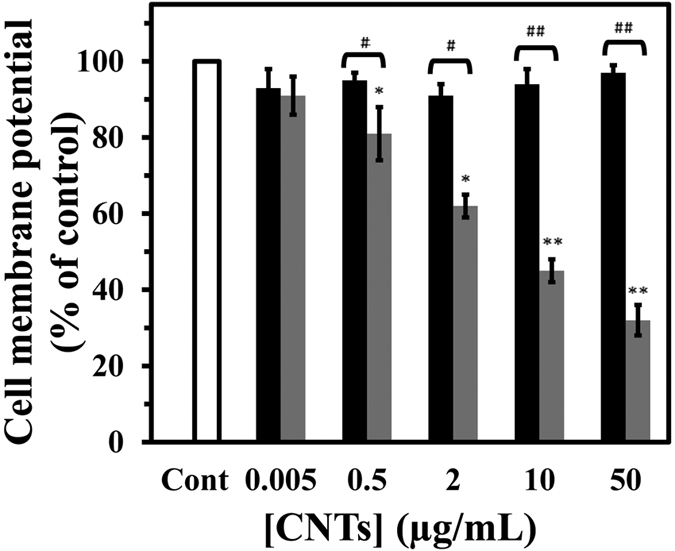
Effect of SWCNT and MWCNT on mitochondrial membrane potential (MMP) in PC12 cells. The PC12 cells were incubated with different concentration of SWCNT (black) or MWCNT (gray) for 48 h. Data are shown as average of three separate experiments and error bars represent standard deviation (SD).*P < 0.05 and **P < 0.01 represents the significant differences between CNTs –treated groups and control. ^#^P < 0.05 and ^##^P < 0.01 represents the significant differences between SWCNT –treated groups and MWCNT –treated groups.

Cell membrane plays a key role to both cell function and the regulation of cell death. Collapse of the cell membrane potential (CMP) is one of the cytotoxicity effects that activate intracellular events involved in induction of necrosis^42^. To evaluate the role of cell membrane in SWCNT and MWCNT-induced necrosis of PC12 cells, the fluorescent anionic dye (DiBAC_4_)was used to examine changes in CMP. When PC12 cells were incubated with various concentrations of SWCNT and MWCNT for 48 h, a significant decrease in the CMP for MWCNT-treated cells was observed. MWCNT dose-dependently caused the more disruption of CMP in PC12 cells in comparison with SWCNT (Fig. 14). At the highest concentration (50 μg/mL), CMP decreased to 3.17 ± 0.22% and 68.41 ± 0.20% for SWCNT and MWCNT, respectively, relative to control. The present data revealed that MWCNTs induces necrosis of PC12 cells accompanied by the alterations in the cell membrane potential, and are in good agreement with the data resulted from caspase activity, staining, flow cytometry, LDH, and MTT assay.

### Effect of SWCNT and MWCNT on mitochondria membrane potential

**Figure 15:**
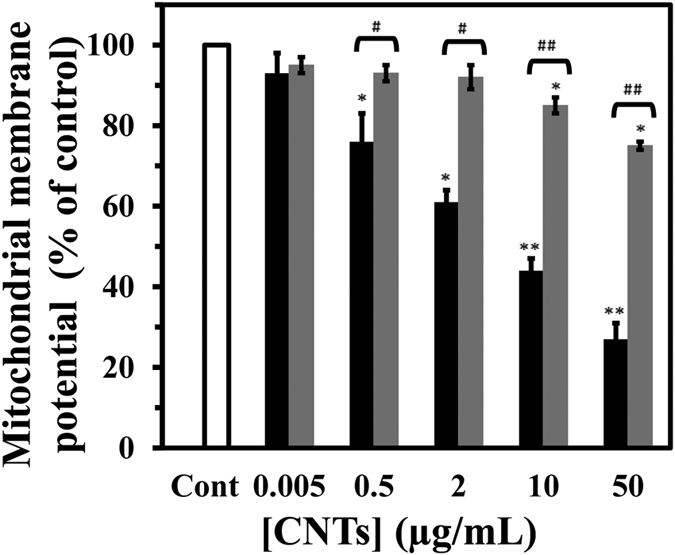
Effect of SWCNT and MWCNT on cell membrane potential (CMP) in PC12 cells. The PC12 cells were treated with raising concentration of SWCNT (black) or MWCNT (gray) for 48 h. Data are shown as average of three separate experiments and error bars represent standard deviation (SD). *P < 0.05 and **P < 0.01 represents the significant differences between CNTs –treated groups and control. ^#^P < 0.05 and ^##^P < 0.01 represents the significant differences between MWCNT –treated groups and SWCNT –treated groups.

Mitochondria, is a double membrane-bound organelle present in most eukaryotic cells, known as energy power plant to support the growth, differentiation and proliferation of cells. The effects of CNTs on mitochondrial activity were examined by investigating their effects on the mitochondria membrane potential (MMP) which is necessary for the regulation of mitochondrial function. Treatment of PC12 cells with SWCNT resulted in a concentration-dependent reduction in MMP. When PC12 cells were incubated with SWCNT for 48 h at highest concentration of 50 μg/mL, MMP decreased to 73.8% of control cells (Fig. 15). There were no significant signs of MMP toxicity (at highest concentration of 50 μg/mL MMP decreased to 24.5% of control cells) in MWCNT treated cells in respect to SWCNT-treated cell. These different outcomes can be considered as the surface polarity differences and avoiding favorable linkage network between mitochondria membrane and MWCNT.

An important issue that was considered in this paper is the influence of the CNTs with different physicochemical characteristics on cytotoxicity which is in accordance with previous studies. Cytotoxicity of lung epithelial cells when exposed to ZnO or TiO_2_ nanoparticles was influenced by their various shapes and forms^43^. Differential inflammatory and cellular responses were reported for spherically shaped or sheets of ZnO nanoparticles^44^. Silver nanorods were seen to induce more toxic response in the human lung epithelial cells than nanospheres with the same concentration^45^. Thus, the influence of the nanoparticles shapes and following surface polarity may be considered when assessing cytotoxic outcomes of SWCNT and MWCNT.

## Discussion

In this research, we explored the effects of CNTs and corresponding hydrophobicity of the surfaces on the structural changes of tau protein and also investigated the effect of CNTs on nervous system *in vitro*. CNTs present unique physicochemical properties which make them attractive in medicine application. However, once they interact with proteins and cells, the effects of CNTs to structural changes of proteins and cells and organelles membranes are not comprehensively understood. In our study, the average charge distribution of the SWCNT and MWCNT remained almost identical when dispersing in PBS. Dispersed SWCNT and MWCNT also exhibited average charge distribution in a closed level in the culture medium. Far UV-CD studies did not show any changes in the content of secondary structure of the tau protein after MWCNT interaction, whereas the spectrum of the tau sample after addition of raising concentration of SWCNT exhibits a significant shift of the minimum to higher wavelengths. Spectral changes of near UV-CD was more pronounced for SWCNT/tau interaction compared to MWCNT/tau, indicating a more significant disruption of the tertiary structure of tau protein in the presence of SWCNT relative to MWCNT. Fluorescence data revealed that SWCNT display the value of K_SV_ higher than MWCNT indicating further evidence for static quenching of SWCNT relative to MWCNT. Cytotoxicity of SWCNT and MWCNT on PC12 cells was also determined by the MTT, LDH, AO/EB staining, flow cytometry, caspase 3 activity, cell and membrane potential assays for monitoring cell viability. MTT assay showed that SWCNT could induce higher *in vitro* cytotoxicity against PC12 cells compared to the MWCNT. However, LDH assay demonstrated that MWCNT has dose-dependent cytotoxicity on PC12 cells while SWCNT exhibited weaker cytotoxicity. As observed microscopically and by flow cytometry, SWCNT exposed to PC12 cells had a substantial increase in apoptotic cells, while MWCNT caused a significant increase in necrotic cells. Moreover, caspase-3 assay demonstrated that SWCNT induced a higher apoptotic rate in PC12 cell compared to MWCNT. These finding reveals that surface tension is a characteristic of nanoparticle hydrophobicity of aqueous solution, and can be used to deduce the nanoparticle-induced alteration of protein structure and cytotoxicity.

Moreover, SWCNT and MWCNT dispersing in PBS and cell culture medium demonstrated almost similar Zeta potential distribution which suggests identical behavior of CNTs such as dispersion and agglomeration in studied solutions. Zeta potential measurement showed that SWCNT and MWCNT had identical Zeta potential value when dispersing in PBS buffer solution. PBS buffer solution with ionic strength of 20 mM, shifts the potential difference between bulk solution and the shear plane toward the bulk solution and the Zeta potential is a relative indicator for the surface charge distribution of the solution instead of particle surface. When CNTs are dispersing in the cell culture medium, Zeta potential of the two kinds of CNTs and cell culture medium was almost identical. This data demonstrated that serum protein molecules of cell culture medium were covered onto SWCNT and MWCNT surface. This data implied that the diameter and charge distribution of CNTs staying in a closed level and do not interfere with different outcomes of interaction with studied biological system such as tau protein and PC12 cell.

Structural differences and more hydrophobic portions of MWCNT relative to SWCNT are the main driving forces for faster adsorption of MWCNT at the air-water interface and eventually lowering the surface tension of solution. Due to presence of hydrophobic surfaces and smaller surface area, MWCNT structures have a less extensive hydration shell than SWCNT^46^. Based on the experimental condition of this study, hydrophobic surface of MWCNT is much more apparent than that on SWCNT surface and results in decreased surface tension of MWCNT solution. This difference allows us to study the influence of the surface polarity of CNTs on the interaction with biological system such as proteins and cells. Tau protein is a dipole with two domains of opposite charge, which the amino-terminal region is negatively charged with a pI of 3.8 followed by the proline-rich domain (pI of 11.4) and the carboxyl-terminal region has a pI of 10.8^47^. Therefore, tau protein has long sequence of positively and negatively charged regions with a concomitant decrease in intermolecular hydrophobic interaction^48^. Increased surface tension of SWCNT implies decreased hydrophobic patches of nanoparticle surfaces which are prone to establish more polar-polar interactions with tau protein relative to MWCNT/tau protein.

As the blood stream is most likely the first entrance of nanoparticles in the human body, understanding of the CNTs interactions with the blood proteins such as fibrinogen (Fg), gamma globulin (Ig), transferrin (Tf), and serum albumin (SA) is essential so that the considerable potential properties of these particles may be safely applied without disruption of protein conformation. Thus, a potential detail of the ruling features modulating the interactions between blood proteins and CNTs is of considerable interest. First, a deep survey of the number of hydrophobic surface residues reveals that there is an order of FG > IG > Tf > SA^49^. These survey and detailed spectroscopic and cellular outcomes of this study both indicate that the blood protein–CNTs interactions may be dictated by hydrophobic residues within each protein. An FG molecule possesses large molecular length, high distribution of hydrophobic residues, and rod like structure maybe bind to MWCNT. It is also subjected to interwinding and forming unfolding native structure onto the surfaces of MWCNT, probably because of hydrophobic-hydrophobic interaction. However Ig, Tf, and SA have a relatively globular structure and lower hydrophobic residues on the surface relative to FG. Therefore unlike FG molecules, three other proteins maybe bind themselves to the surface of the SWCNT by more hydrophilic interaction mediated by water molecules. Indeed, our analysis may demonstrate that the π–π stacking interactions between CNTs and aromatic residues (Trp, Phe, and Tyr) play a pivotal role in their binding potentials. In general, it is more likely for a protein to be interacted by a protein if it provides the almost identical surface tension values with nanoparticle solution. The selective attachment of blood proteins on the CNT surface may affect the nanoparticle assembly and cellular responses and cause to different cytotoxicity. These outcomes might reveal more details into our comprehension of how to design and develop the safe nanoparticles in medicinal applications.

**Figure 16:**
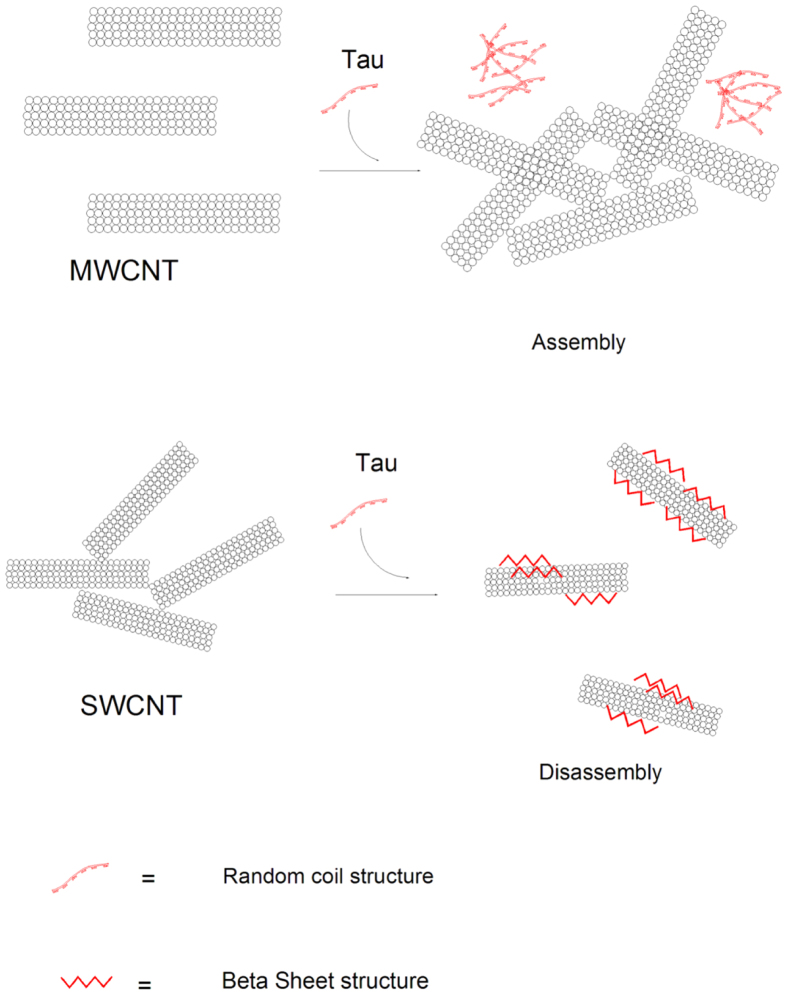
Schematic representation of interaction of SWCNT and MWCNT with tau protein. Tau protein is attached on the surface of SWCNT and demonstrates structural changes from random coil structure to β-sheet structure. Also after absorption of protein, disassembly of SWCNT is observed. MWCNT does not alter the structure of tau protein and MWCNT is susceptible to self assembly in presence of tau protein.

**Figure 17:**
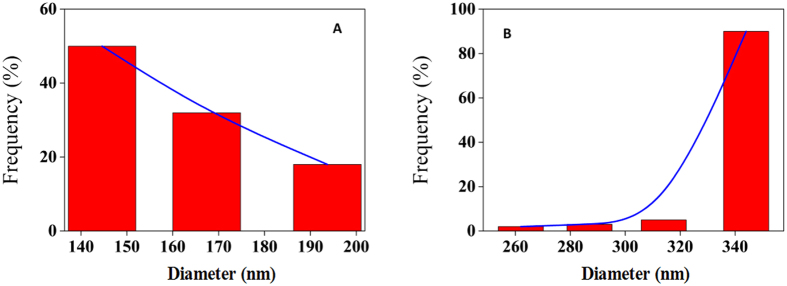
Particle size measurement. Particle size measurement of MWCNT in the absence (**A**) and presence (**B**) of tau protein using DLS.

Based on the surface tension study, the hydrophobic surfaces of MWCNT were more exposed to solution molecules compared to SWCNT. This state is thermodynamically unfavorable, and agglomeration occurs to form a self-assembled state of MWCNT to stabilize it and perform a reduction of MWCNT-tau protein interaction. Conformational changes of protein and agglomeration of CNTs are dependent on their nature surface tension. Hydrophobic MWCNT dispersed in hydrophilic tau protein media often tend to agglomerate. Hence, the interactions between a tau protein and CNTs are a mixture of different interactions such as electrostatic forces, van der Waals attraction, hydrogen bonding and hydrophobic effects. In the case of MWCNT, favorable interaction could not be established between protein and nanoparticle surfaces owing to their polarity differences. Therefore, MWCNT is susceptible to self-assembly and agglomeration in presence of tau protein which can be called protein assisted nanoparticle self-assembly (PANSA) (Fig. 16). To seek further supports for this hypothesis DLS and TEM sample analyses were carried out to determine the average size of the samples and visualize the dispersion of CNTs. To determine if tau protein could induce aggregation of MWCNT, we designed an interaction strategy and compared average size of individual sample (MWCNT) to that of a combination of samples (MWCNT and tau protein). When MWCNT used individually the hydrodynamic radius of MWCNT was observed to be ~160 nm by DLS (Fig. 17A). However, the interaction of MWCNT with tau protein resulted in drastic aggregation enhancement of sample with a hydrodynamic radius of ~341 nm by DLS (Fig. 17B).

**Figure 18:**
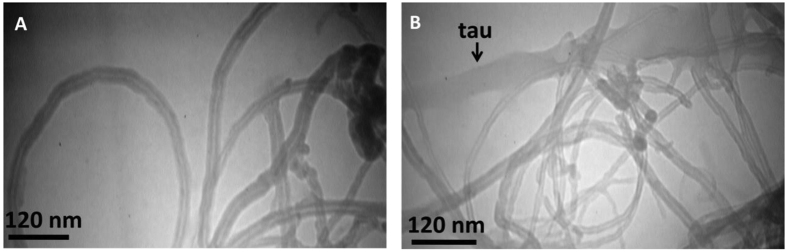
TEM observation. TEM image of MWCNT in the absence (**A**) and presence (**B**) of tau protein. MWCNT is susceptible to self-assembly in presence of tau protein and also tau protein has been aggregated upon titration of MWCNT.

TEM shows formation of tau aggregates and bundle aggregates of MWCNT of various diameters which are grown upon interaction with tau in regards to MWCNT alone (Fig. 18A,B).

Full-length tau protein yields a pI of 8.61 which arises from the combination of basic and acidic residues and at pH 7.8, the net charge and isoelectric point for full-length tau protein is assigned around 2 and 8.6, respectively. It was also demonstrated only the C-terminal tail of tau protein is speculated to contain considerable hydrophobic helical structure^32^. In this study at pH 7.8, the zeta potential of tau protein and CNTs samples was approximately 1 and −4, respectively. Interaction mechanism is suggested to may depend on charge distribution over the nanoparticle and protein surfaces. However, in the case of interaction of negatively charged CNTs and positively charged tau protein, the magnitude of charge distribution is not high enough for establishing electrostatic interaction. Therefore it seems that MWCNT due to lower surface tension and corresponding higher hydrophobic residues would prefer to attach itself to the hydrophobic patches of tau which is placed on the C-terminal residues. However this interaction is not a potential candidate in establishing hydrophobic - hydrophobic interactions between protein and MWCNT surfaces.

**Figure 19:**
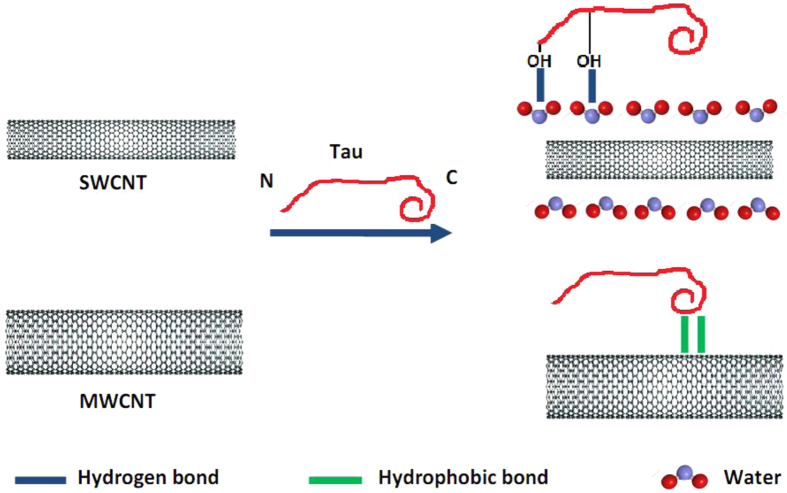
Schematic representation of different binding site and binding forces of tau protein upon interaction with SWCNT and MWCNT. The binding site for the absorption of tau protein onto the SWCNT surface will be N-terminal domain by means of hydrogenic bonds which is mediated by water molecules. However, C-terminal residues of tau protein are attached to the MWCNT surface by hydrophobic-hydrophobic interactions.

**Figure 20:**
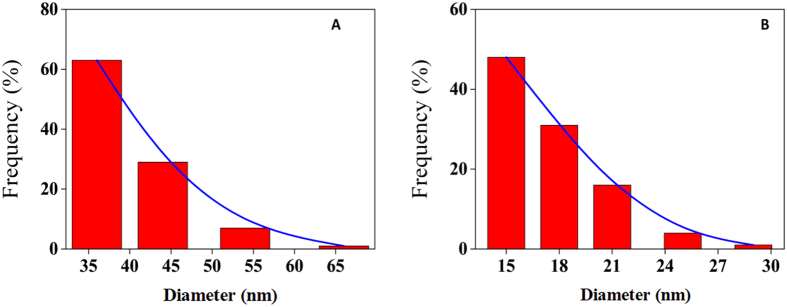
Particle size measurement. Particle size measurement of SWCNT in the absence (**A**) and presence (**B**) of tau protein using DLS.

Regarding SWCNT, π-π interactions are also dominant forces among nanotubes and induce SWCNT aggregation. However in the presence of tau protein, due to their almost identical surface tension value, it may be concluded that hydrogen interactions are being established between SWCNT and tau surfaces. Indeed, protein and CNT surfaces are surrounded by water, and tau protein binds SWCNT in the presence of water that drives hydrogen bond formation between these species. Hence, the binding site for the interaction of tau protein and SWCNT will be N-terminal domain with high distribution of hydrophilic residues (Fig. 19). To more support this conclusion, we examined the effect of the tau protein on SWCNT assembly also by using DLS and TEM. DLS measurement showed the SWCNT have hydrodynamic radius of 40 nm (Fig. 20A) but when the SWCNT brought to interaction with tau protein, it presents a hydrodynamic radius of 17 nm (Fig. 20B) which has been disentangled from aggregation.

**Figure 21:**
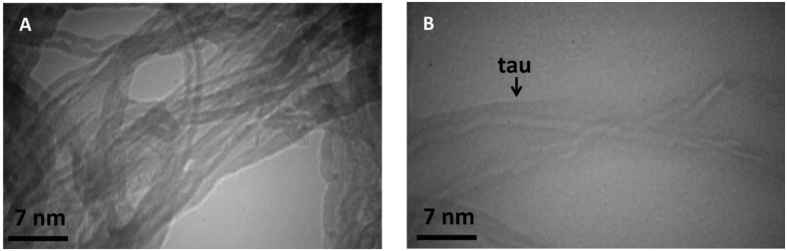
TEM observation. TEM image of SWCNT in the absence (**A**) and presence (**B**) of tau protein. Tau protein is absorbed on the surface of SWCNT and demonstrates disassembly of SWCNT.

TEM images of the SWCNT alone revealed bundle-aggregated SWCNT (Fig. 21A). With the addition of tau protein to the SWCNT solution, the size of the aggregated SWCNT decreased and it dispersed with a thin layer of adsorbed tau protein on its surface (Fig. 21B). These interesting results are in good agreement with spectroscopic data as well.

Based on these data which were supported by a variety of techniques, a new theory as the Falahati-Saboury theory may be proposed. This theory expresses that the surface tensions of protein and nanoparticle can play a pivotal role in the interaction mode between nanoparticles and proteins. In other words, if protein and nanoparticles carry almost identical surface tension values then protein-nanoparticle interaction dominates the protein-protein or nanoparticle-nanoparticle interaction and subsequently prevents particle aggregation.

It can be concluded that protein topology and its structural surface polarity can affect the CNTs assembly.

Accordingly, SWCNT and MWCNT showed to have different tendencies to induce conformational changes of tau protein in accordance with the definition of “hydrophilic” and “hydrophobic” interactions, respectively. In general, SWCNT reported to have higher surface tension, and can perform a wider range of induced conformational changes in the tau protein. Therefore, structural changes of proteins with the interaction of nanoparticles are not always occurring and/or dependent on protein and the nanoparticle surface polarity. In this regard, the hydrophobic portions of nanoparticle and protein surfaces are linearly correlated to the extent of protein conformational changes. The hydrophobic portions are non-existent especially at the extreme cases of conformational changes, where protein exists as a linear polypeptide chain as a native form. Given that we observe a different influence of surface tension on CNTs-induced conformational change of protein, must arise from driving forces of the nanoparticle–protein interaction process. Interaction can be considered as a two step complex formation; the first step is the tau protein establishes new networks with the CNTs surface via van der Waals forces and adsorbs loosely in the native form at random orientation on CNTs surface. Then, the tau protein can undergo new and additional linkages to form stronger non-covalent protein–CNTs bonds which result in nanoparticle-induced protein conformational changes^50^,^51^. Therefore, the non-covalent binding and following reorientation of protein on CNTs surface can result in lateral interactions between the adsorbed proteins and following intermolecular protein interactions and production of a heterogeneous protein population. The significant SWCNT-induced conformational changes of tau protein are therefore determined by the balance of the hydrophilic coverage on the surfaces of tau protein and SWCNT. Based on the main driving force for the interaction of tau protein with high content of available hydrophilic residues in the protein with CNTs, hydrophilic surfaces of SWCNT is essential for the interaction process. Furthermore, given the limited conformational changes of tau proteins on hydrophobic MWCNT surface, the charge distribution of the tau protein surface presumably may not be changed and corresponding repulsive electrostatic interactions can play an important role on the avoiding protein-protein interactions. On the other hand, protein–protein repulsions are geometrically reduced due to the enhanced hydrophilic surface in SWCNT, causing a denser protein layer and heterogeneous population of protein. This hypothesis is supported by CD and fluorescence experiments which demonstrated the initial conformational change of tau protein occurs at very low concentration for SWCNT indicating that for hydrophilic nanoparticles, contribution of the lateral repulsions is considerably small. Additions of high concentrations of SWCNT and MWCNT in solutions containing tau proteins can lead to aggregation, due to the formation of intermolecular interactions by the protein (near-UV CD and fluorescence section). Thus the fate of structural changes of protein upon interaction with nanoparticle surface are both governed by their own chemical properties and nanoparticle surface polarity. Varying the nanoparticle shape would most likely alter protein structure and heterogeneity by modifying the density of absorbed proteins on SWCNT and MWCNT surfaces. It can be suggested that beyond an optimum surface tension, the apparent tau protein-CNT interaction increases owing to contribution of polar-polar forces. In addition, it can be concluded that the extent of protein heterogeneity upon interaction with nanoparticle directly correlates with CNTs and protein surface tensions. The surface tension of underlying CNTs could influence the orientation of the absorbed tau protein so that CNTs-tau protein binding becomes more heterogeneous. With interaction of tau protein with SWCNT, the higher structural changes and the following more heterogeneous population of absorbed protein occurred. These results suggest that the extent of heterogeneity in the protein-nanoparticle interaction is directly related to the surface polarity of host and absorbed spices. These differences in protein stability after interaction with SWCNT and MWCNT may have been referred to the types of protein interaction and protein binding.

In the present study also anti-proliferative activity of SWCNT and MWCNT on PC12 cells were studied. A principal question that was taken into consideration in this research is the effect of the CNTs with different physicochemical features on cytotoxicity which is in consistence with previous studies^43^,^44^,^45^. Our results demonstrated that SWCNT presented the stronger growth inhibitory effects on the viability of PC12 cells compared with MWCNT. The amount of SWCNT required to reach IC_50_ at 48 h treatment was considerably lower than that of MWCNT. Although results of MTT assay clearly demonstrated that SWCNT is comparatively more cytotoxic than MWCNT against PC12 cells, LDH activity assay showed that SWCNT exhibited weaker cytotoxicity effects. These results might reveal different characteristic properties of CNTs which SWCNT can increase the apoptotic effects, while MWCNT can increase the necrotic induction against PC12 cells. This is in consistence with dual AO/EB staining, flow cytometry analysis, and caspase-3 assay that demonstrated SWCNT induced a higher apoptotic rate against PC12 cells compared to MWCNT. Cell membrane damage can result in activation of signaling cascades contributing to necrotic cell death. In this study, we also investigated effects of SWCNT and MWCNT on MMP and CMP of PC12 cells. SWCNT exerted a decrease in MMP; while MWCNT did not induce significant effect on MMP. Therefore, loss of MMP probably also participated in SWCNT induced apoptosis of PC12 cells. Comparing intensity of membrane potential of SWCNT and MWCNT-treated cells revealed that MWCNT apparently affects the cell membrane potential relative to SWCNT and MWCNT switched on necrosis mode of PC12 cells.

**Figure 22:**
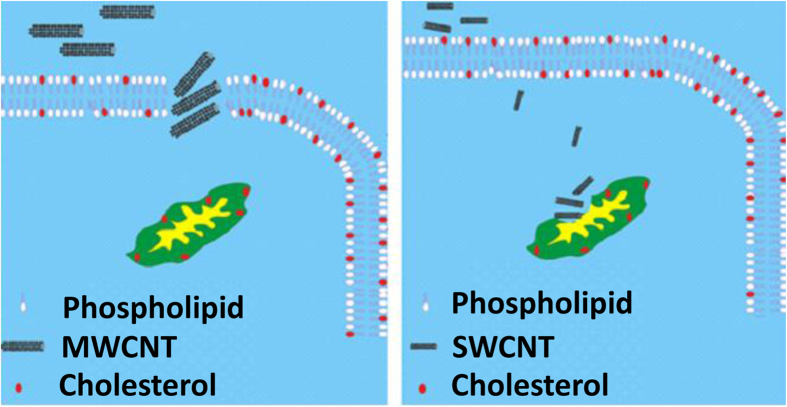
Schematic representation of interaction of SWCNT and MWCNT with cell membrane and mitochondria. Cell membrane shows higher cholesterol distribution than mitochondria membrane and therefore provides more hydrophobic microenvironment. MWCNT with lower surface tension results in membrane integrity, whereas SWCNT with greater surface tension leads to mitochondria damage.

Almost all types of nanoparticles including dendrimers, vesicles, micelles, core–shell, microbubbles and CNTs have been evaluated for their imaging, drug delivery and cytotoxicity^52^,^53^. However, the exact mechanisms by which nanoparticles exert their cytotoxic effects and mode of action in cells remain unknown. Cellular toxicity is usually governed in addition to size and charge by another characteristic such as surface tension. Surface tension of nanoparticles influences their target binding, dispersion and agglomeration. Therefore, cytotoxicity could also be defined owing to different kind of interaction between target molecules and CNTs. On the other hand, biophysical parameters such as surface tension of CNTs, cells and organelles membranes types also influence cytotoxicity. In this context, due to higher hydrophobic portion and lower surface area, MWCNT structures have a less hydration shell than SWCNT and may cause hydrophobic-hydrophobic interaction of MWCNT with the cell membrane and following hole formation and loss of the plasma membrane integrity. Cell membranes possess typical average of 20% cholesterol in the whole membrane and up to 50% in rafts (−% is molecular ratio)^54^. Daum^55^ also reported that the cholesterol content of membrane plasma is 30 μg per mg of protein, whereas mitochondria contain around 3 μg per mg of protein^55^. These non identical distributions of cholesterol content may induce microenvironments with different hydrophobicity for mitochondria and cell membranes (Fig. 22). In other words, the increased cholesterol content of biological membrane is correlated with the hydrophobicity elevation of the system. Hence, it could be concluded that cell membrane is more prone to form “hydrophobic-hydrophobic” contacts with MWCNT than mitochondria membrane. At interaction of mitochondria membrane and MWCNT “hydrophobic-hydrophobic” contacts are almost absent, while they are frequent in all the interaction of MWCNT with cell membrane. MWCNT can also bind with membrane-bound proteins which have hydrophobic surfaces via hydrophobic-hydrophobic interactions. MWCNT provides a high affinity to membrane proteins via nonpolar-nonpolar interactions and its binding may change protein stability and interfere with membrane integrity. However, no obvious mitochondria membrane damage of PC12 cells was reported for MWCNT. This different sensitivity of plasma and mitochondria membranes to surface polarity of CNTs may lead to surface tension-dependent modes of cytotoxicity which can be called (STDMC). Different degrees of hydrophobicity of CNTs, cells and mitochondria membranes may be a descent reason for the disparate outcomes. The surface hydrophobicity of MWCNT and cell membrane is speculated to the increased hydrophobic-hydrophobic interaction and corresponding damage which caused by a higher hydrophobicity of MWCNT and cell membrane compared to SWCNT and mitochondria membrane. A number of features indicate that hydrophilic effect is likely to be an important factor in the interaction between mitochondria membrane and SWCNT. First a large number of negative charge of phospholipids and corresponding polar surface area of mitochondria membrane is exposed to water. The surface tension measurements revealed that SWCNT introduces a more hydrophilic surface and hydration shell in comparison with MWCNT particles. It appears that MWCNT with lower surface tension causes membrane damage, whereas SWCNT with greater surface tension results in intracellular damage. MWCNT acted more by disruption of membrane integrity, whereas SWCNT induced apoptosis to a greater extent in PC12 cells (Fig. 22). In this regards, CNTs can translocate intact into the cell without being aggregated or they can be aggregated near the cell or organelle membranes based on membrane and nanoparticle characteristics^56^,^57^,^58^,^59^. Interaction of CNTs with mitochondria membrane depends on their physicochemical properties such as surface tension. Interaction of MWCNT with lower surface tension (relative to SWCNT) and corresponding higher hydrophobicity and mitochondria membrane with higher hydrophilicity (relative to cell membrane) results in less favorable interactions and energy contribution and corresponding agglomeration of MWCNTs. In other words, surface hydrophobicity of both membrane and CNTs is crucial factor for determining absorption and interaction. The hydrophilicity of SWCNT is helpful for binding in a polar-polar interaction manner with the hydrophilic mitochondria membrane. Therefore the surface tension of nanoparticle can affect their induced cytotoxicity.

## Conclusion

The outcomes of our research proposes that the interaction of CNTs must be assessed before they are used as a carrier for drug delivery to nervous system widely, because structural changes of tau protein and viability of neuron cells will cause neurodegenerative disease.

## Materials and Methods

### Materials

CNTs were purchased from Neutrino Co. (Tehran, Iran). The cell culture medium (RPMI 1640), penicillin–streptomycin, fetal bovine serum (FBS) and horse serum were purchased from Gibco BRL (Life technology, Paisley, Scotland). The culture plates were obtained from Nunc Co. (Denmark). Ethidium bromide (EB) and acridine orange (AO) were purchased from Pharmacia LKB Biotech Co. (Sweden). MTT (3-(4,5-dimethyltiazol-2-yl)-2,5-diphenyltetrazoliumbromide), nerve growth factor (NGF) and dimethyl sulfoxide (DMSO) were purchased from Merck Co. (Darmstadt, Germany). Annxexin V-FITC apoptosis detection kit (ApoAf-50TST) was purchased from Sigma-Aldrich Company, St. Louis, USA. PC12 cells line (rat adrenal pheochromocytoma cells) were purchased from pasture institute of Tehran, Iran. All other materials were purchased from Sigma-Aldrich Company, St. Louis, USA.

### CNTs dispersion and characterization

CNTs were dispersed in ethanol for characterization study and in order to reduce agglomeration, the stock suspensions (200 μg/mL) were mixed using vortex for 5 min, and sonicated for 60 min using a sonicator probe(Misonix- S3000, USA) at 30 °C. The stock concentration was then serially diluted with ethanol and sonicated as before to use for titration.

Ultraviolet- visible (UV–vis) spectra of SWCNT and MWCNT with a concentration of 0.4 mg/mL in ethanol were monitored in 1 cm quartz cuvettes using a Shimadzu UV–vis spectrophotometer at a scan rate of 10 nm S^−1^ over the range 190–800 nm and the intensity and position of wavelength maximum was determined. Also the morphology of CNTs was investigated with the aid of the scanning electron microscope [(SEM) (TESCAN vega3, Czech Republic)]. Later, the CNTs dispersions were analyzed using TEM (Zeiss - EM10C - 80 KV, Germany) to reveal the size of the individual CNTs.

### Dynamic light scattering

CNTs were dispersed in 20 mM phosphate buffer solutions pH 7.8 (for protein structural studies) and in cell culture medium (for cytotoxicity studies). The behavior of suspended CNTs in PBS and cell culture medium was analyzed using dynamic light scattering [(DLS)(Malvern Instrument, Worcestershire, United Kingdom)]with an argon ion laser set at excitation wavelength of 488 nm, and a fixed 90° scattering angle. DLS and Zeta potential measurement were performed for CNTs samples in the absence and presence of tau protein. Prior to DLS and Zeta potential experiments, the samples go through sonication for 5 min.

### Surface tension measurement

Tau protein was first dissolved in 20 mM phosphate buffer solutions at pH 7.8. Surface tension assay of tau protein, SWCNT and MWCNT with a concentration of 0.1 mg/mL were measured with a Kruss K100 tensiometer using Du Noüy Ring method at room temperature. The measurements were done for each sample in triplicate, and the average was used for data analysis. All measurements were subtracted with PBS and cell culture solutions.

### Circular dichroism measurement

The far (190–250 nm) and near (240–320 nm) UV-CD studies were done by an Aviv model 215 Spectropolarimeter (Lakewood, New Jersey, USA). Dry nitrogen gas was supplied to purge the equipment before and during the course of the measurements. The data was expressed as a molar residue ellipticity [θ], defined as [θ] = 100 θ_obs_MRW/CL, where θ_obs_ is the observed ellipticity in degrees, C is the tau protein concentration in mg cm^−3^, MRW is the mean residue weight and L is the length of the light path in cm. Circular dichroism (CD)spectra were recorded with a time constant of 2 s, a 1-nm bandwidth, a scan rate of 10 nm min^−1^ and a 1-mm path length cell from 250 to 190 nm for secondary and from 320–240 nm for tertiary structural changes of protein. CD samples were prepared with a concentration of tau protein (200 μg/mL for far UV-CD and 500 μg/mL for near UV-CD) and different concentration of CNTs [(0, 10, 50 and 100 μg/mL) for far UV-CD] and [(0, 5, 10, 50, 40, 60, 80 and 100 μg/mL) for near UV-CD] were used. All spectra were also subtracted with CNTs solutions and were smoothed.

### Fluorescence spectroscopy measurements

Intrinsic fluorescence intensity of tau protein was measured using Carry Eclipse (Varian, Australia) spectrofluorimeter on the excitation wavelength of 274 nm and emission wavelengths 307 nm. Emission and excitation slits were set to 5 and 10 nm, respectively. The structural changes of tau protein (50 μg/mL) were monitored in the presence of different concentrations of SWCNT and MWCNT (0, 5, 10, 20, 50 and 100 μg/mL) at room temperature. The mixture of tau protein with CNTs was vortexed for 5 s and then incubated for 1 min to form protein-CNTs complex before measurement of the fluorescence intensity. All spectra were subtracted with CNTs solutions and were smoothed.

### Cell culture

PC12 cell line (rat adrenal pheochromocytoma cells, obtained from Pasteur Institute, Tehran, Iran) was cultured in DMEM medium, supplemented with FBS (10%), penicillin (100 units/mL) and streptomycin (100 μg/mL). PC12 cells were grown in CO_2_ incubator (Memmert, Germany) at 37 °C with 90% humidity and 5% CO_2_. The cells were detached regularly using trypsin/EDTA and cultured in a new cell culture flask. The cells were differentiated by adding nerve growth factor (NGF; 100 ng/mL) for 6 days.

### Cultivation of PC 12 cells with different concentration of SWCNT and MWCNT

A defined number of cells (1 × 10^6^ cells/well) in a 96-well plate were seeded and after 24 h different concentration of SWCNT and MWCNT suspensions (0.01, 0.5, 2, 10, 50 and 100 μg/mL) was added to the wells and viability of the cells was determined after 48 h by the [(3-(4,5-dimethylthiazol-2-yl)-2,5-diphenyl-tetrazolium-bromid (MTT)] assay.

### MTT assay

The antiproliferative effects of SWCNT and MWCNT were measured using MTT colorimetric assay. Briefly, PC12 cells were seeded in 96-well microplates at a density of (1 × 10^6^ cells/mL), and after 24 h of cell attachment, plates were washed twice with phosphate buffered saline (PBS) and then cells were cultured in medium with varying concentrations (0.01, 0.5, 2, 10, 50 and 100 μg/mL) of SWCNT and MWCNT for 48 h. PC12 cells in culture medium without SWCNT and MWCNT were designated as reference blanks. Ten μl of MTT (5 mg/mL in PBS) was added to each well and the plates were incubated 37 °C for 4 h in a 5% CO_2_ incubator. The medium was then carefully removed, and the purple products were lysed in 200 μl DMSO. The plates were vibrated for 15 min and the absorbance, which was proportional to cell viability was measured at 570 nm using a microplate reader (Expert 96, Asys Hitch, Ec Austria). Cytotoxicity was expressed as a percentage of growth inhibition, relative to the control culture value, and the concentration of CNTs required to inhibit PC12 cell growth by 50% (IC_50_) was calculated. Each experiment was repeated in triplicate to calculate the standard error, and finally statistical analysis was performed using the student’s -test.

### Measurement of cell membrane integrity

PC12 toxicity by CNTs was assayed by measuring the cytosolic enzyme activity, lactate dehydrogenase (LDH), released into the culture medium following the membrane leakage induced by CNTs (0.1, 1, 4, 10 and 50 μg/mL). Samples from clarified medium of treated and untreated groups were taken after 48 h of incubation, and LDH activity was determined by spectrophotometrically (Expert 96, Asys Hitch, Ec Austria) monitoring the reduction of pyruvate according to the manufacturer’s protocols (Parsazmoon, Tehran, Iran). LDH release was calculated as the percentage of LDH in the medium versus total LDH activity in the cells. All experiments for LDH assay were repeated in triplicate and data are represented as the mean ± SD.

### Acridine orange and ethidium bromide double staining

Cell morphology was examined under a fluorescence microscope (Zeiss, Germany) by staining the cells with DNA-binding dyes acridine orange (AO) and ethidium bromide (EB) (Sigma, St. Louis, Mo, USA). After being incubated in media containing IC_50_ concentration of SWCNT and MWCNT for 48 h, the cells were fixed in 4% formaldehyde (w/v) for 40 min and washed three times with PBS and cell nuclei were stained with a mixture of AO (100 μg/mL) and EB (100 μg/mL) at room temperature for 10 min. Finally, apoptotic DNA fragmentation of PC12 cell treated with CNTs was investigated. evaluation of apoptotic and necrotic cells was then assessed for both treated and untreated PC12 cell which is characterized by living cells with normal green nucleus, apoptotic cells with bright green and orange-stained nucleus (condensed or fragmented chromatin) and necrotic cells with uniformly orange-stained cell nuclei.

### Flow cytometry analysis

For flow cytometry analysis, PC12 cells were seeded in 96-well microplates at a density of 1 × 10^6^ cells in the absence and presence of SWCNT and MWCNT with a single concentration of IC_50_ of SWCNT and MWCNT. After 48 h, cells were centrifuged at 1000 rpm for 5 min and the cells were collected and washed twice with PBS buffer. After that, the cells were resuspended in 500 μl of binding buffer followed by addition of 10 μl of Annexin V-FITC and 20 μl of propidium iodine (PI) and were incubated for 5 min in the dark place at the ambient temperature. Finally light scattering were analyzed using the flow cytometer (BD Biosciences FACS CalibureTM) with a flowing software 2.5.1, equipped with a argon ion laser, and a band pass filter (FL-1 channels) for fluorescence maximum excitation/emission: Annexin V, FITC: 494 nm/518 nm; PI: 535 nm/617 nm.

### Caspase-3 activation assay

The extent of caspase-3 activation in PC12 cells incubated with SWCNT and MWCNT (0.1, 1, 4, 10 and 50 μg/mL) was assessed using commercially available kit (Sigma, St. Louis, Mo, USA). PC12 cells were cultured (1 × 10^6^ cells/mL) and incubated with SWCNT and MWCNT for 48 h. At the end of the incubation, the cell lysates for caspase-3 assay were prepared by the application of lysis buffer supplied with the kit and incubated with caspase-3 substrate (N-acetyl-DEVD-p-nitroaniline) at 37 °C for 2 h in 96-well culture plates. Finally, absorbance in wells was measured at 405 nm using a microplate reader (Expert 96, Asys Hitch, Ec Austria). The increase in the activity of caspase-3 was compared with values of control cells.

### Measurement of cell membrane potential

The slow-response potential-sensitive fluorophore, DiBAC_4_^3^ (Bis-(1,3-Dibutylbarbituric Acid) (TrimethineOxonol) (B-438, Invitrogen) was used to measure the relative cell membrane potential (CMP) upon incubation with CNTs (0.005, 0.5, 2, 10 and 50 μg/mL). PC12 cells were incubated with 50 nMDiBAC for 30 min at 4 °C, and finally washed three times with PBS. After incubation, the cells were centrifuged at 1,000 g for 2 min and supernatants were assayed for fluorescence intensity studies. The changes in fluorescence intensity of DiBAC_4_^3^ were monitored by using a flow cytometer (BD Biosciences FACS CalibureTM) at the excitation wavelength of 490 nm and emission wavelength of 516 nm. The amount of DiBAC_4_^3^ fluorophore which partitions into the cells was inversely proportional to fluorescence intensity of the DiBAC_4_^3^ remaining in the supernatant.

### Measurement of mitochondrial membrane potential

The changes in mitochondrial membrane potential (MMP) of PC12 cells were calculated using cationic fluorophore, Rhodamine 123 (Rh 123). Treated and untreated cells exposed to different kinds of CNTs (0.005, 0.5, 2, 10 and 50 μg/mL) were washed and suspended in ice-cold PBS. Afterwards, cells loaded with 15 mM Rh 123 at 4 °C for 30 min and then washed three times with PBS. After incubation, the cells were centrifuged at 1,000 g for 2 min and the supernatant was removed and undergoes fluorescence intensity analysis by a flow cytometer (BD Biosciences FACS CalibureTM) at the excitation and emission wavelength of 485 nm and 530 nm, respectively. This method gives ratiometric, inversely proportional values of MMP and dye in supernatant.

### Statistical analysis

All the experiments were performed in triplicate and data are shown as mean ± SD. Statistical analysis was performed according to the Student’s t -test and one-way ANOVA analysis followed by Dunnett’s multiple comparison tests. The probability values of P < 0.05 were considered as significance.

## Additional Information

**How to cite this article**: Zeinabad, H. A. *et al*. Interaction of single and multi wall carbon nanotubes with the biological systems: tau protein and PC12 cells as targets. *Sci. Rep.* **6**, 26508; doi: 10.1038/srep26508 (2016).

## Figures and Tables

**Table 1:** Properties of SWCNT and MWCNT powder.

	Purity	OD (nm)	ID (nm)	SSA (m^2^/g)	Making method
SWCNT	>90%	1–2	0.8–1.6	>380	CVD
MWCNT	>95%	10–20	5–10	>200	CVD

Data were given based on the manufacturers’ reported values. Outer diameter (OD), Inner diameter (ID), specific surface area (SSA).

**Table 2:** Properties of SWCNT and MWCNT in solution.

	OD (nm)	Average size in PBS	Average size in cell culture
SWCNT	1–2	40.6 ± 5.9	61 ± 4.6
MWCNT	10–20	160.8 ± 19.5	171 ± 21.7

SWCNT and MWCNT were sized using DLS in PBS and cell culture medium. Data were compared with manufacturers’ reported outer diameter (OD).

**Table 3:** Zeta potential of SWCNT and MWCNT in solution.

	Average Zeta potential of cell culture medium	Average Zeta potential in PBS	Average Zeta potential in cell culture medium
SWCNT	−17.7 ± 2.5	−4.4 ± 1.7	−18.6 ± 3.4
MWCNT	−17.1 ± 3.1	−4.1 ± 1.2	−17.5 ± 2.9

Distribution of the Zeta potential of SWCNT and MWCNT in solution recorded using the dynamic light scattering technique.
