# Erratum: Interaction of single and multi wall carbon nanotubes with the biological systems: tau protein and PC12 cells as targets

**DOI:** 10.1038/srep29644

**Published:** 2016-09-21

**Authors:** Hojjat Alizadeh Zeinabad, Alireza Zarrabian, Ali Akbar Saboury, Ali Mohammad Alizadeh, Mojtaba Falahati

Scientific Reports 6: Article number: 2650810.1038/srep26508; published online: 05 24 2016; updated: 09 21 2016

This Article contains discrepancies between the HTML and PDF versions of Figures 14 and 15. The correct Figures 14 and 15, with their accompanying legends appear below as Figures 1 and 2 respectively.

## Figures and Tables

**Figure 1:**
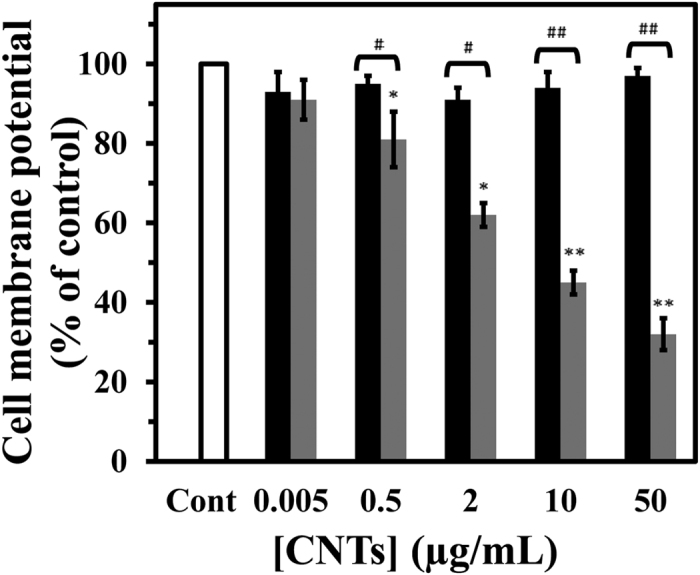
Effect of SWCNT and MWCNT on cell membrane potential (CMP) in PC12 cells. The PC12 cells were incubated with different concentration of SWCNT (black) or MWCNT (gray) for 48 h. Data are shown as average of three separate experiments and error bars represent standard deviation (SD). ^*^P < 0.05 and ^**^P < 0.01 represents the significant differences between CNTs –treated groups and control. ^#^P < 0.05 and ^##^P < 0.01 represents the significant differences between SWCNT –treated groups and MWCNT –treated groups.

**Figure 2:**
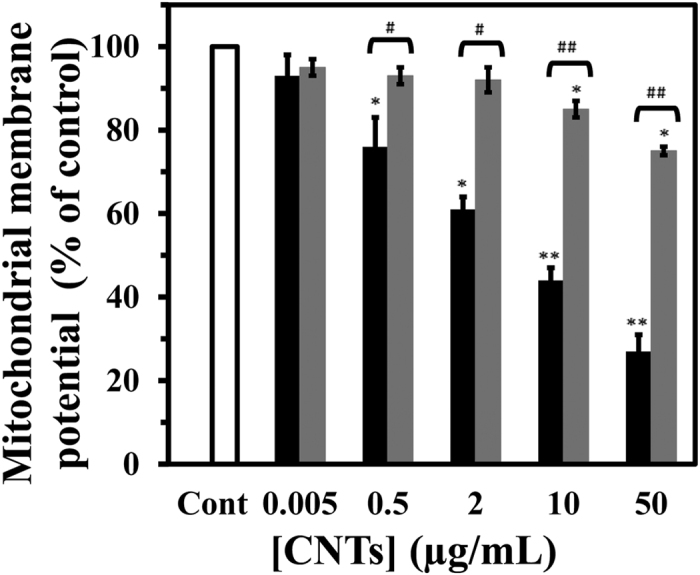
Effect of SWCNT and MWCNT on mitochondrial membrane potential (MMP) in PC12 cells. The PC12 cells were treated with raising concentration of SWCNT (black) or MWCNT (gray) for 48 h. Data are shown as average of three separate experiments and error bars represent standard deviation (SD). ^*^P < 0.05 and ^**^P < 0.01 represents the significant differences between CNTs –treated groups and control. ^#^P < 0.05 and ^##^P < 0.01 represents the significant differences between MWCNT –treated groups and SWCNT –treated groups.

